# Antimicrobial resistance pattern in domestic animal - wildlife - environmental niche via the food chain to humans with a Bangladesh perspective; a systematic review

**DOI:** 10.1186/s12917-020-02519-9

**Published:** 2020-08-24

**Authors:** Shahneaz Ali Khan, Mohammed Ashif Imtiaz, Md. Abu Sayeed, Amir Hossan Shaikat, Mohammad Mahmudul Hassan

**Affiliations:** 1 Chattogram Veterinary and Animal Sciences University, Zakir Hossain Road,Khulshi, 4225 Chattogram, Bangladesh; 2 Jhenaidah Government Veterinary College, Jhenaidah, Bangladesh

**Keywords:** AMR, Domestic animals, Wildlife, Insects, Environment, Food chain, Bangladesh

## Abstract

**Background:**

Antimicrobial resistance (AMR) is a growing concern globally, but the impact is very deleterious in the context of Bangladesh. Recent review article on the AMR issue demonstrates the scenario in human medicine; unfortunately, no attempt was taken to address this as One Health issue. The antimicrobial resistance bacteria or genes are circulating in the fragile ecosystems and disseminate into human food chain through direct or indirect ways. In this systematic review we are exploring the mechanism or the process of development of resistance pathogen into human food chain via the domestic animal, wildlife and environmental sources in the context of One Health and future recommendation to mitigate this issue in Bangladesh.

**Results:**

Tetracycline resistance genes were presenting in almost all sample sources in higher concentrations against enteric pathogen *Escherichia coli*. The second most significant antibiotics are amino-penicillin that showed resistant pattern across different source of samples. It is a matter of concerns that cephalosporin tends to acquire resistance in wildlife species that might be an indication of this antibiotic resistance gene or the pathogen been circulating in our surrounding environment though the mechanism is still unclear.

**Conclusions:**

Steps to control antibiotic release and environmental disposal from all uses should be immediate and obligatory. There is a need for detailed system biology analysis of resistance development *in-situ*.

## Background

Antimicrobial resistance (AMR) is one of the emerging issues globally including in low and middle-income countries (LMICs) for human health threats [1]. Primarily, AMR is the ability of a microbe to avoid the effects of an antimicrobial even though exposure to recommended doses. AMR in bacteria can be achieved by several ways including the inherent capability of natural resistance by certain bacteria, genetic mutation or acquired resistance through their surroundings [2]. Bangladesh is a developing and middle-income country in Southeast Asia (SEA) with a dense human population. The presence of diverse wildlife as well as a livestock population (cattle, sheep, goat, and poultry etc.) reservoir, has identified Bangladesh as a country where high prevalence of antibiotic resistance is documented against important pathogens in humans [3]. Developing countries such as Bangladesh are vulnerable to AMR issues for their poor surveillance health care facilities [4], unhygienic and unregulated conditions of the agriculture, livestock and aquaculture food production process, poor sanitation, widespread misuse and irrational antibiotics and prophylactics use in poultry, livestock and aquaculture industry [5]. The probiotics used in veterinary feeds contribute to the burden of antibiotic resistance bacteria (ARB) and/or antibiotic resistance genes (ARGs) in the human food chain [6]; therefore, people in the community acquiring resistance pathogens from food, environment, and wildlife sources. People affected with various illnesses (for example urinary tract infection) may not respond to the first line of drugs (such as amoxicillin, amoxiclav, ampicillin, and ciprofloxacin) available for their treatment [3]. This enhances the risk of mortality, longer duration of hospitalization and higher hospital costs. Additionally, physicians may resort to alternative drugs such as fosfomycin, nitrofurantoin, tigecycline, carbapenems [7].

Poultry production system is considered a high risk for AMR emergence in low-income settings, particularly in Bangladesh, where commercial poultry production is rapidly increasing. The majority of antimicrobial classes are used both in humans and animals (such as domestic mammals, birds, and farmed fish etc.) [8]. There are significant differences in the ways of treating companion animals (dog, cat, pet birds) compared to food-producing animals (poultry, cattle). In the case of food animals, entire flocks or pens are treated with antimicrobials through feed and water [9]. In addition, food animals exposed to long term, low dose, mass medication for the purpose of growth promotion create a favorable condition for selection and spread of resistant bacteria within and between groups of animals and humans [10]. Moreover, the non-veterinarian (popularly called *Quack*) prescribed antibiotics, used unwisely and unprofessionally for treating diseases in animals overwhelmed this resistance pattern.

ARB and ARGs dissemination from food producing animals to the surrounding environment niches, takes place in the excretion of antimicrobials through urine or feces onto surface waters and soils, or the application of animal manure as fertilizer to soil or ponds [11]. Untreated animal waste is used for a variety of purposes in subsistence economies like Bangladesh. Movement of food and animals has also led to the development of global dissemination of AMR.

Environmental contamination with antibiotic residues and resistant organisms/genes due to human activity has been demonstrated from pharmaceutical plants, hospital effluents and untreated wastewater, and may be a leading driver of ABR in low-resource settings [12].

Little is known about the ecology of AMR outside human and animal hosts; however, we increasingly understand that by focusing only two of these One Health compartments of the transmission circle, will result in an incomplete epidemiological background of resistance mechanism [9]. Bacterial populations are significantly diverse, both originating from aquatic and soil habitats. This poses the serious possibility of acquiring resistance capability through selection pressure from their environment which can be transmitted to humans either by direct contact with animals or food products, or indirectly via environmental pathways [9]. Recently a review article entitled “Antibiotic resistance in Bangladesh: A systemic review” has been published by AMR regarding issues in human medicine [3]. Unfortunately, no attempts have yet been made regarding the AMR issue in domestic animals, wildlife and the environment. Additionally, the human food chain is the major route of transmission of ARB and ARGs from other sources [9]. In this review, we discuss this issue systematically. The goal was to generate reference for future works and provide a recommendation to negotiate the AMR through implementing a One Health program.

## Result

### Demographic characteristics of different studies utilised in this review

We reviewed 45 articles that described AMR in different samples from domestic animals, wildlife, food sources (mainly originated from animal sources), environmental and insects. The majority of the articles published (53%) during the recent period (2015–2019), highlights the importance of the AMR issue in Bangladesh (Table 1). Most of the research work is based in Dhaka and surrounding cities (Mymensingh, Savar), due to the ease of both collecting samples and access to laboratories for testing.

**Table 1:** Characteristics of the studies included in the review (*N* = 45)

Characteristics	Frequency (%; 95%CI)	Reference	
**Publication Year**			
2005–2010	2 (4.44; 0.05–15.14)	[13, 14]	
2011–2014	19 (42.22; 27.65–57.84)	[15–33]	
2015–2019	24 (53.33; 37.87–68.33)	[34–57]	
**Location**			
Dhaka	13 (28.88; 16.36–44.31)	[13, 14, 20, 22–24, 30–32, 34, 36–38]	
Chittagong	6 (13.33; 5.05–26.79)	[15, 21, 39–42]	
Rajshahi	2 (4.44; 0.05–15.14)	[17, 43]	
Mymensingh	10 (22.22; 11.20-37.08)	[19, 26, 28, 35, 44–47, 55, 56]	
Dinajpur	2 (4.44; 0.05–15.14)	[27, 48]	
Savar	2 (4.44; 0.05–15.14)	[18, 33]	
Kustia	1 (2.22; 0.05–11.77)	[49]	
Dhaka + Mymensingh	1 (2.22; 0.05–11.77)	[50]	
Chittagong + Rajshahi	1 (2.22; 0.05–11.77)	[51]	
Rajshahi + Hakaluki	1 (2.22; 0.05–11.77)	[52]	
Gazipur + Savar	1 (2.22; 0.05–11.77)	[16]	
Gazipur + Cox’s bazar	1 (2.22; 0.05–11.77)	[53]	
Cox’s bazar + Kuakata	1 (2.22; 0.05–11.77)	[29]	
Chittagong + Cox’s bazar + Rangamati	1 (2.22; 0.05–11.77)	[25]	
Mymensingh + Gazipur + Sherpur	1 (2.22; 0.05–11.77)	[54]	
Jamalpur + Tangail + Netrokona + Kishorganj	1 (2.22; 0.05–11.77)	[57]	
**Type of sampling source**			
Live bird market	6 (13.33; 5.05–26.79)	[13, 28, 34, 39, 54, 57]	
Fish market	2 (4.44; 0.05–15.14)	[47, 56]	
Egg market	3 (6.66; 1.39–18.66)	[14, 21, 22]	
Dairy farms	3 (6.66; 1.39–18.66)	[35, 44, 55]	
Poultry farms	4 (8.88; 2.47–21.22)	[16, 18, 19, 33]	
Scavenging/ Household farm/ Backyard/Wild birds	6 (13.33; 5.05–26.79)	[17, 25–27, 45, 48]	
Water/ River/ Household water	3 (6.66; 1.39–18.66)	[32, 43, 52]	
Street food + Ready to eat food + Street food vendor/ Supershop/ Restaurant	8 (17.77; 8.05–32.05)	[20, 23, 24, 36, 37, 40, 42, 49]	
Veterinary hospital/ Medical/ Slaughter house and or effluents/dustbins	7 (15.55; 6.49–29.45)	[15, 30, 31, 38, 46, 50, 51]	
Wild life	3 (6.66; 1.39–18.66)	[29, 41, 53]	
**Sample Category**			
Chicken	10 (22.22; 11.20-37.08)	[13, 16–19, 33, 34, 39, 54, 57]	
Food	5 (11.11; 3.70-24.05)	[20, 36, 37, 40, 56]	
Egg	3 (6.66; 1.39–18.66)	[14, 21, 22]	
Milk	5 (11.11; 3.70-24.05)	[23, 24, 35, 44, 55]	
House crows	2 (4.44; 0.05–15.14)	[41, 51]	
Pigeon + Duck + Quail	6 (13.33; 5.05–26.79)	[25–28, 45, 48]	
Brown headed gulls	1 (2.22; 0.05–11.77)	[29]	
Open bill stork	1 (2.22; 0.05–11.77)	[52]	
Wild ducks	1 (2.22; 0.05–11.77)	[17]	
Dog + Cat + Cattle	2 (4.44; 0.05–15.14)	[38, 50]	
Deer	1 (2.22; 0.05–11.77)	[53]	
House flies	2 (4.44; 0.05–15.14)	[46, 47]	
Cockroach	1 (2.22; 0.05–11.77)	[42]	
Household water/ effluents/ water	5 (11.11; 3.70-24.05)	[15, 30–32, 43]	
Currency	1 (2.22; 0.05–11.77)	[49]	
**Pathogen isolated**			
* E. coli*	16 (35.55; 21.86–51.21)	[13, 16, 17, 25, 26, 29–32, 39, 43, 46, 49, 51–53]	
* Salmonella*	13 (28.88; 16.36–44.31)	[14, 18, 19, 21, 22, 27, 28, 40, 41, 45, 47, 48, 56]	
Enterobacter	1 (2.22; 0.05–11.77)	[33]	
* Staphylococcus*	5 (11.11; 3.70-24.05)	[15, 38, 42, 50, 55]	
* E. coli + Salmonella*	3 (6.66; 1.39–18.66)	[35, 37, 54]	
* E. coil + Staphylococcus*	1 (2.22; 0.05–11.77)	[44]	
* E. coli + Salmonella + Staphylococcus*	2 (4.44; 0.05–15.14)	[34, 36]	
* E. coli + Salmonella + Staphylococcus + Vibrio*	1 (2.22; 0.05–11.77)	[23]	
* E. coli + Salmonella + Campylobacter*	1 (2.22; 0.05–11.77)	[57]	
* E. coil + Staphylococcus + Klebsiella*	1 (2.22; 0.05–11.77)	[24]	
* Staphylococcus + Alcalegene + Klebsiella + Enterococcus + Actinobacillus + Proteus*	1 (2.22; 0.05–11.77)	[20]	
**Sample category *** (N = 48)*			
Domestic	6 (12.5; 4.72–25.26)		
Food	15 (31.3; 18.65–46.25)		
Environment	14 (29.2; 16.95–44.06)		
Wildlife	7 (15.6; 6.07–27.76)		
Insects	6 (12.5; 4.72–25.26)		

There are also good number of articles published based on the samples collected from other two cities (Rajshahi and Chittagong) (Table 1). The prominent source of samples were collected from live birds markets, scavenging poultry farming, commercial poultry farms and super shops or street food or restaurant (Table 1). If we consider the sample category, the most number of samples were collected from poultry including layer and broilers, pigeon [11]. There are several wildlife species that were sampled including deer, brown headed gulls, house crow, open bill stork and wild duck. There are a few studies that included wildlife environmental samples such as water bodies under the roosting site of wild birds (open bill storks) and hospital effluent wastage (house crow). Several food sources including vegetables, eggs and milk were included in several articles, in order to determine the resistance pattern against major zoonotic pathogen. Overall; 15 articles described the resistance patterns against *Escherichia coli* isolated from different sources of samples and there were also 12 articles for *Salmonella* spp. We observed that in 45 papers, they described 48 sample sources and we divided these into 5 categories. The highest number of samples (31.3%) was from food sources and 29.2% were from environmental sources. We also found similar percentages (12–15%) of samples were from domestic animals, wildlife and insect species.

### Meta-analysis revealed the prevalence of*E. coli* and *Salmonella spp.* isolated from different source of samples.

**Fig. 1:**
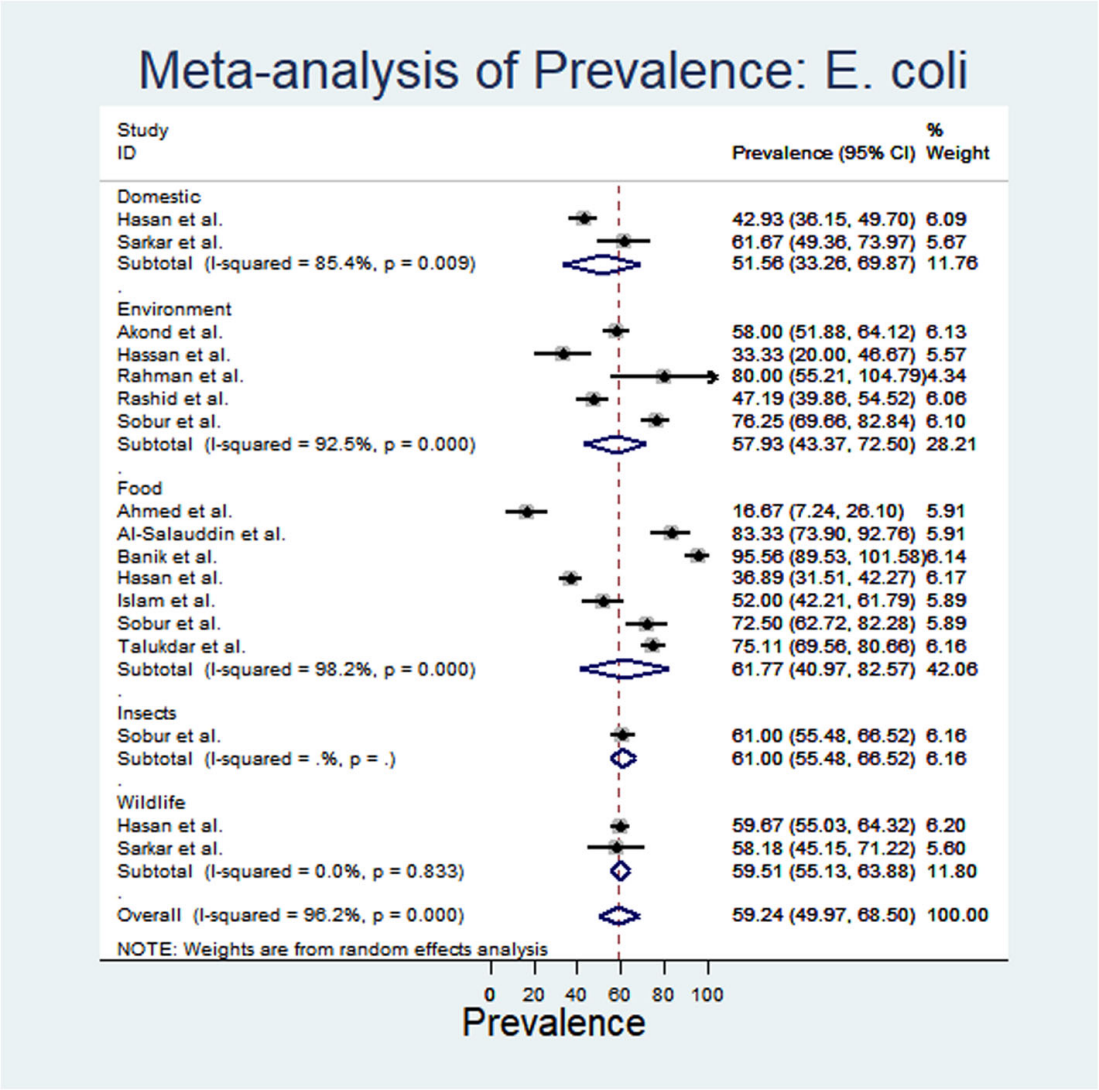
Forest plot of *E. coli* prevalence isolated from different samples (the center dot representing point estimates where as Gray Square representing the weight of each study to the meta-analysis)

**Fig. 2:**
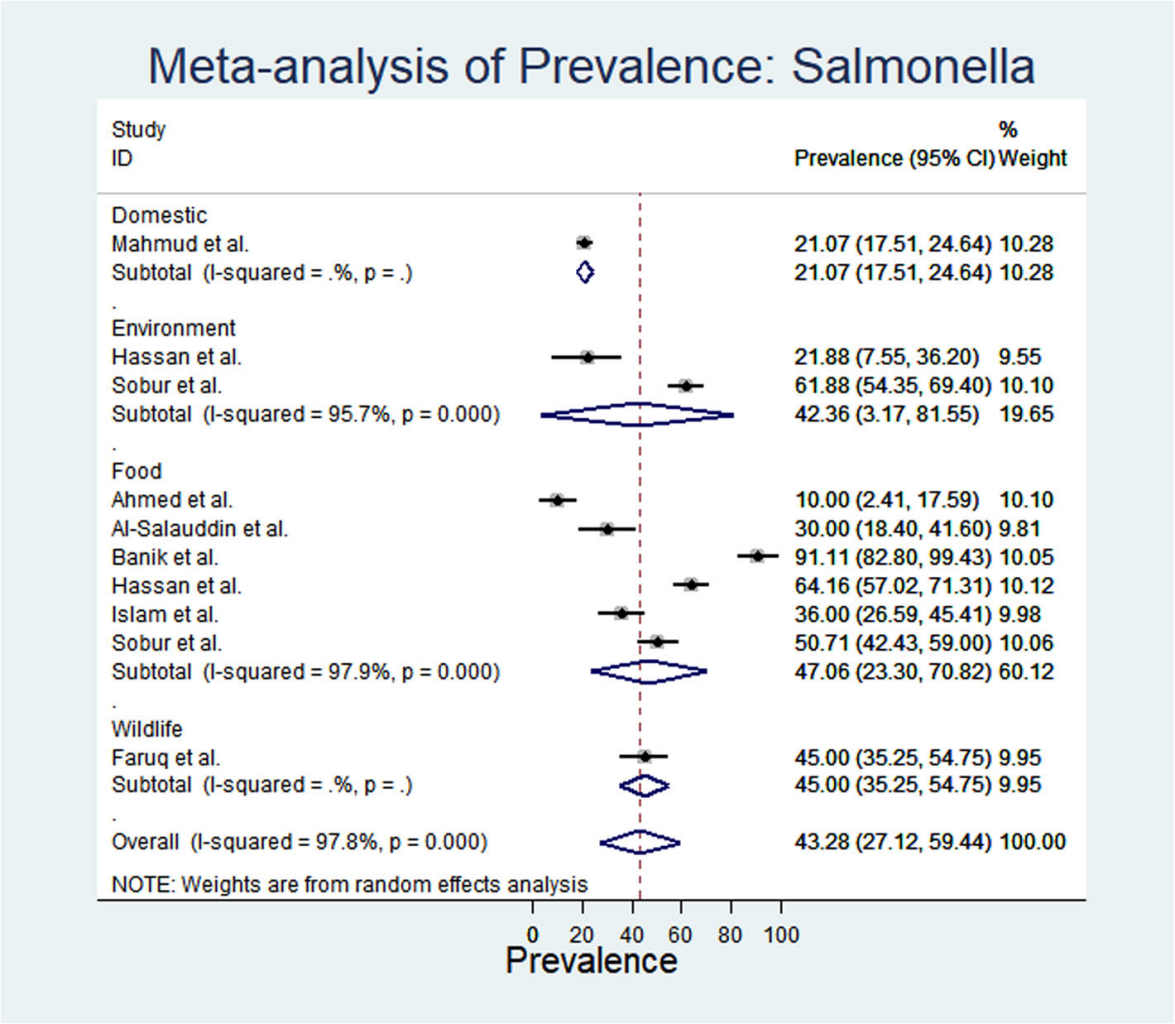
Forest plot of *Salmonella* spp. prevalence isolated from different samples (the center dot representing point estimates whereas gray square representing the weight of each study to the meta-analysis)

From meta-analysis, the estimated prevalence of *E. coli* ranged from 16.67% (95% CI: 7.24–26.1) to 95.68% (95% CI: 95.68 -89.53-101.58) with substantial heterogeneity (I^2^: 96.2%) in different sample categories (Fig. 1). The random effect estimated pooled prevalence was 59.24% (95% CI: 49.97–68.59). Overall, the highest prevalence of *E. coli* was recorded from food sources 61.77% (95% CI: 40.97–82.57) followed by insects (61%), wildlife (59.51%), environment (57.93%) and domestic animals (51.58%) (Fig. 1). Again, the estimated prevalence of *Salmonella* spp. ranged from 10% (95% CI: 2.41–17.59) to 91.11% (95% CI: 82.8-99.43); whereas, the heterogeneity varied from 21.07–47.06% in different sampled categories (Fig. 2). Moreover, the prevalence of salmonellosis varied from 21.07% in domestic animals and 42.36%, 45% and 47.06% in environmental, wildlife and food sources respectively.

### The antibiotic resistance prevalence percentage for *E. coli* and *Salmonella* spp.

**Fig. 3:**
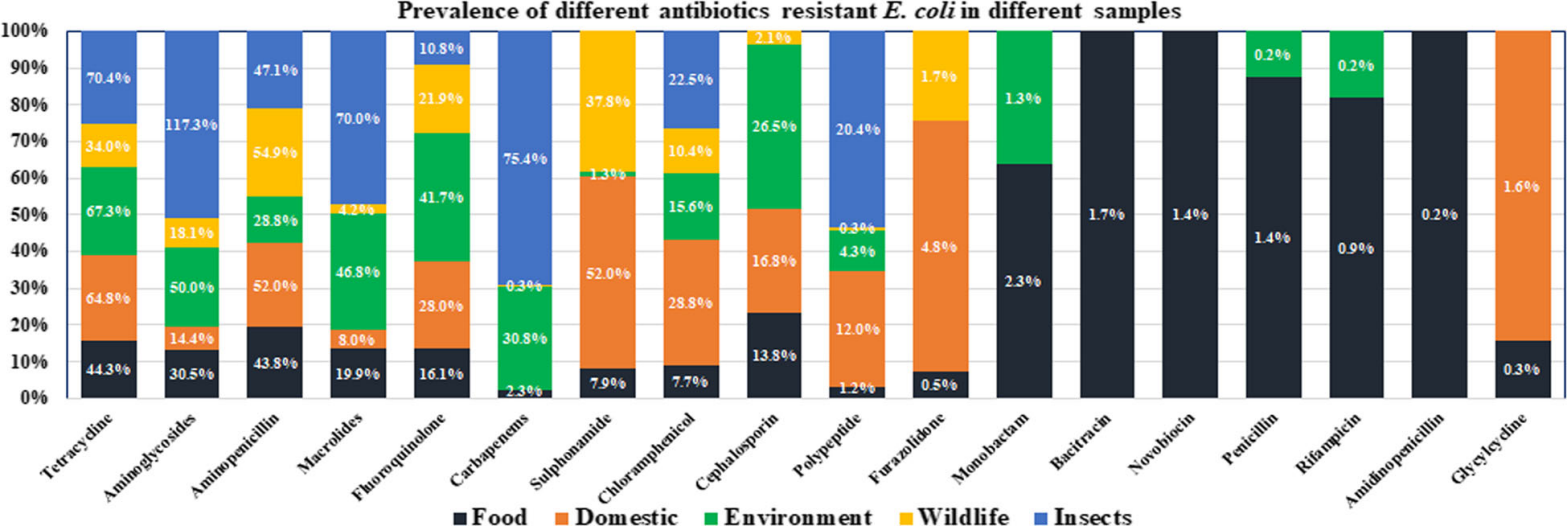
The 100% Stacked bar chart illustrating antibiotics resistance prevalence against *E. coli* in contrast to different sample category (some of the values in the bar are > 100% due to repeated count of the antibiotics against each sample because each sample may be resistant against more than 1 antibiotic of same genera)

**Fig. 4:**
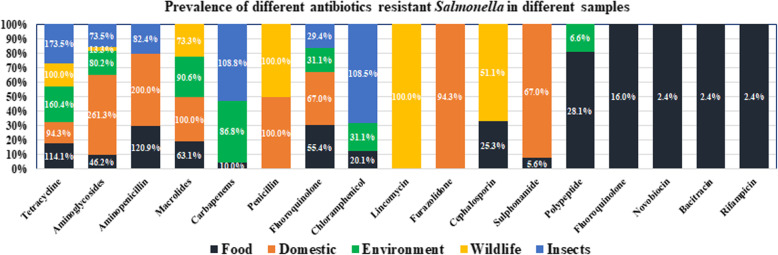
The 100% Stacked bar chart illustrating antibiotics resistance prevalence against *Salmonella* spp. in contrast to different sample category (some of the values in the bar are > 100% due to repeated count of the antibiotics against each sample because each sample may be resistant against more than 1 antibiotic of same genera)

Among all the antibiotics tetracycline, aminoglycosides, amino-penicillin, macrolides, fluroquinolone, chloramphenicol and polypeptides were resistant at different degree varied from 0.3–117.3% in all five sample categories in contrast to *E. coli* (Fig. 3). Besides, for *Salmonella* spp., only tetracycline and aminoglycosides were resistant at wider range 13.3 to 261.3% (Fig. 4). Across the different groups of sources, tetracycline resistance ranged from 34 to 70.4% against *E. coli*, whereas the range varied 94.3–173.5% in *Salmonella* spp. For amino-penicillin the resistance percentage was around 47–200% (*E. coli* 54.9% on highest and *Salmonella* spp. 82.4 to 200%) in samples collected from wildlife, food sources, domestic animals and also insects. The environmental samples showed 28% against *E. coli*.

The highest percentage of prevalence was demonstrated in food samples, domestic animal samples, environmental and wildlife samples. We observed a very low percentage of resistance against several antibiotics including polypeptide, furazolidone, monobactam, glycylcycline, nobvobiocin and bacitracin. For chloramphenicol, only insects (houseflies) showed a higher percentage (108%) of resistance. It is alarming that cephalosporin is as high as 51% in wild birds. Macrolides are useful for animal and human and their highest percentage of resistance was observed in wild life (51%) and insects (76%).

## Discussion

**Fig. 5:**
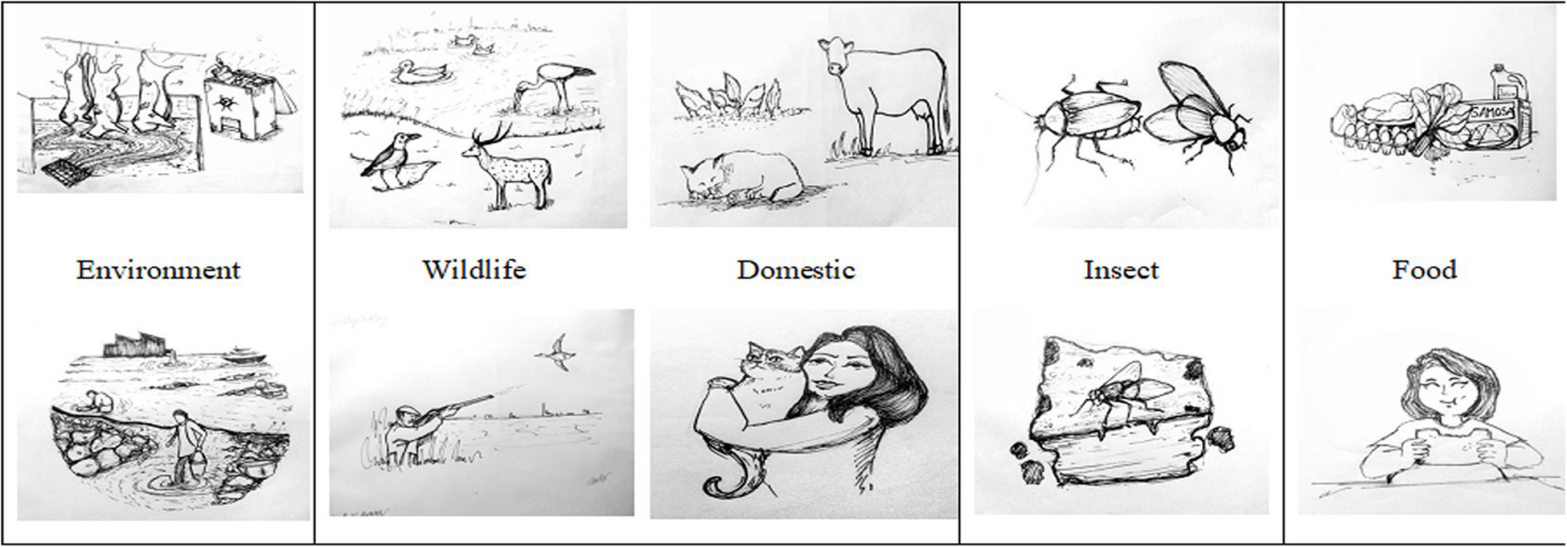
Transmission dynamics of antimicrobial resistance bacteria or genes into human food chains through different components of One Health

Though AMR is a human health issue, but this phenomenon been developed at the human-animal-wildlife-environmental interface and subsequently the resistance gene or the bacteria get entry to human food chain (Fig. 5). Hence the One Health issue is a significant concept to get insights of this AMR problem in this systematic review paper in the context of Bangladesh.

The majority of the AMR study was conducted in large cities namely Dhaka, Chittagong, Rajshahi, and Mymensingh. There are several veterinary and research laboratory facilities in these parts of the country. Unfortunately, there is no systemic and structured surveillance on AMR issues, although it is a global concern. Considering antibiotic resistance patterns, it is very alarming that cephalosporin is highly resistant in wild animal species [58, 59], although it is unclear how this resistance occurs in those species. Bangladesh is a country of diverse wildlife and migratory bird species providing a unique environment for wildlife and domestic animal interaction; posing a serious impact on developing resistant bacteria. The most popular drug for treating domestic animal is tetracycline and its resistance prevails in all types of samples including food, domestic animals, wildlife and environment; therefore, we need to explore judicial alternatives of tetracycline for livestock production. Amino-penicillin also showed tremendous resistance patterns in all categories sampled. This drug is utilized in human and animal medicine and has highlighted the chance of a resistance gene or bacteria evolving through the interaction of domestic animals, wildlife and the environment directly into the human food chain. Here we have discussed systematically how antibiotic resistance is developing through different sources of One Health components that interplay with each other in the human food chain.

### Resistance pathogen originated from human food

The transmission of resistant bacteria or resistant genes into the human food chain can occur directly or indirectly and in different ways. In this review we found antibiotic resistance of *E.coli* and *Salmonella* spp. in foods including meat (poultry), milk and milk products (both raw and pasteurized) [23, 44], eggs [21, 22], vegetables, ready to eat or street food (singara, lemon juice, fuska, sugarcane juice, borhani etc.) [20, 37, 40] and vegetables [36]. In addition, across all categories of sampling, *E. coli* and *Salmonella* spp. prevalence was higher in the human food chain than that originated from animals. However, Abattoir workers, poultry farm workers [13], vendors in live bird markets [14], food handlers as well as the consumers, represent the large number of people directly at risk of acquiring ARB via the food chain in developing countries where bio-security and food safety measures are limited. The evolving resistance pathogen enter to humans with animals and biological substances (such as blood, urine, feces, milk, saliva, semen) and enhances the rapid transmission and risk of dissemination of resistant bacteria from host to host [4]. Some studies also demonstrated the presence of the residue of different antibiotics (ciprofloxacin, enrofloxacin, amoxicillin, doxycyline, oxytetracylcine, and tetracycline) in poultry meat [60], milk, eggs [61] and fishes [62] as well.

Recently the government of Bangladesh has announced plans to allow meat imports from Brazil and three other countries. This would pose a serious risk for disseminating antibiotic resistance globally and from continent to continent. In addition, Bangladesh imports different food items from around the world which would have a high risk of development of AMR into the human food chain. Recent reports show a high prevalence of plasmid mediated mcr-1 *E. coli* in fecal samples from chicken imported from European countries in Tunisia [63]. This raises concerns as ARB and ARGs emerging in food animals and products in developed nations could endanger food safety and public health care settings in developing countries, where prevention and containment of ABR are limited and where high levels of ABR prevail [6].

### Resistance pathogen originated from domestic animals

The dissemination of active antibiotics, metabolites or degradation products of antibiotics, (antibiotic residues), as well as ARGs and ARB excreted via food animals waste, have established hot spots for important reservoirs of ABR in farm premises and surroundings [4]. Dairy [35] and poultry farms [16, 28] and their environment, such as manure, waste water and vegetables [35] have been identified as hot spots of ABR pollution with antibiotic residues being detected in these areas across the world. The high prevalence of these residues in various ecological niches on the farms exacerbates the issue of AMR in developing countries like Bangladesh, as it increases the pool of ARB and AGFs in the ecosystem. Metagenomics analysis of faecal samples from cattle, swine, and chickens reveal the abundance of resistance genes regardless of antibiotic regime as prophylactics or treatment. Agricultural or host-associated metagenomes harbor an increased abundance of resistance genes encoding resistance against antibiotic and toxic compounds [12]. Horizontally transferred resistance bacteria and genes have been documented in environmental niches such as manure, water and soil, leading to microbial communities containing various levels of ARB. The environmental pollution may therefore, lead to the emergence and spread of new ARB and AGFs in food products [6].

### Resistance pathogen originated from environment and insects

There are two main mechanisms, mutation and acquisition of resistance genes [2], responsible for developing resistance pathogens and the majority of ARGs have originated in the environmental microbiota. Antibiotic use with suboptimal doses, provides favorable conditions for the microbe’s acquisition of resistant gene transfer and recombination in environments, such as farms, hospitals, poultry markets, fish markets and sewage systems, which provide ideal incubation periods for resistance gene acquisition [64].

Antibiotics are not only used for human therapy, but also for farming systems and the surplus antibiotics that are not utilized can go into the dumping process which could have massive ongoing environmental effects. Moreover, some pharmaceutical companies dump antibiotics into the sewerage system, which ultimately end up in the river systems producing a massive impact on resistance strains enabling them to mutate and evolve into super bugs [12]. Unfortunately, millions of metric tons of antimicrobials have been released into the biosphere over the last half century. It is demonstrated that 50 kg ciprofloxacin is being dumped into the river every day in Hyderabad and this is a worst case example of irresponsible disposal [65].

Antibiotics are widely used in the agriculture production chain including use as growth promoters, prophylaxis or therapeutic treatments in aquaculture, pet animals and pest control. Biocides are used in toiletries, hand care and household cleaning products and all of these antibiotics are ultimately dispersed into the human food chain [12]. Together with the antibiotics used in clinics, this can lead to antibiotic contamination on farms, rivers receiving wastewaters and land receiving antibiotic-contaminated manure occurring frequently [66]. This can accelerate evolution towards resistance and also increases the risk for its transfer to human pathogens, which can be present in these ecosystems and this would constantly generate selection and maintenance pressure towards all sorts of resistance strains [67]. In this review, it is demonstrated that hospital and veterinary clinical wastage [51] and slaughterhouse effluents [15] provide a unique environment where bacteria re-assortment and mutation occur through gene transfer to wild (House crow and Asian pied sterling) [51] and domestic animals (scavenging poultry) [16] and inset (houseflies) [46].

### Resistance pathogen originated from wildlife

Bangladesh is a densely populated country with diverse biodiversity and wildlife populations. Wildlife interaction with domestic animals and their environmental niche provide an avenue for developing and disseminating resistance pathogens in a variety of ways. Wild birds as such brown headed gulls and open bill storks living in water bodies where their fecal droppings contaminate rivers [29, 52], might transfer resistant bacteria or genes to other wildlife species, such as scavenging ducks and chickens [52]. They have great opportunities to disseminate resistance bacteria to domestic and scavenging birds and farm environments, where bio-security is arrested or compromised in Bangladesh perspectives. In addition, house crows are dominant scavenging wild birds in Bangladesh and their presence is abundant around dustbins, and effluents of veterinary, medical clinics and slaughterhouses [51]. Crows along with human and animal wastes figure hotspots for bacterial re-assortment, mutation and the emergence of resistant pathogens.

A number of bacteria cause disease in humans and other hosts including livestock and wildlife species and therefore their presence are ubiquitous in the environment. The interplay of this ecologically important pathogen is very crucial for evolution of AMR reservoir microorganism. Wild animals foraging in the human-influenced environment are colonized by bacteria with clinically important antibiotic resistance. The occurrence of such bacteria in wildlife is influenced by various biological, ecological, and geographical factors which are not yet fully understood [64]. A particular concern regarding the role of wild migrating birds in the dispersal of AMR bacteria is their capacity for long-range movements across borders or continents and from regions with high levels of AMR bacterial contamination to less affected areas. This issue is very important in the Bangladesh perspective, where migratory wild birds frequently travel long distances from Siberia to Bangladesh through Australasian pathways. In several studies, the *E. coli*-producing extended-spectrum beta-lactamases that have contributed resistance to third generation cephalosporins, have been identified in wild birds (Brown headed gulls and open bill stork) in remote areas with low human exposure such as in Hakaluki haor, Cox’s Bazar Beach [29].

### One health approach to mitigate the AMR issue

The implications of these findings are global, though this study only covered Bangladesh. A great majority of antimicrobial classes are used in both humans and animals including domestic animals, birds, and farmed fishes [68]. For companion animals, antimicrobial uses are broadly similar to those in humans. In the case of food animals, antimicrobials are frequently used in food and water to the entire group for a prolonged period of time and often at sub-therapeutic doses. These conditions favor the selection and spread of resistant bacteria within and between animals as well as to humans through food consumption and other environmental pathways [69]. There is an increasing concern that heavy metals and biocide poisoning in animals and the environment may contribute to the development of resistance pathogens [36, 70]. In particular, common enteric pathogens with zoonotic importance as such *E. coli*, *Salmonella* spp. and *Campylobacter* spp., shedding through the fecal route, could contaminate meat and poultry products directly or indirectly through fruits and vegetables via environmental contamination [71]. *Salmonella* spp. resistance to any medically important antimicrobial is of public health concern, but is particularly important to human health of cephalosporins and fluoroquinolones [72] resistance, for which therapeutic options can be limited. Beta lactams such as third generation cephalosporins, may often be the only option available to treat serious infections. Concerns have been expressed that fluroquinolone use in food animals is linked to quinolone resistance against *Salmonella* spp. [73]. Many *Salmonella* spp. strains are also resistant to antimicrobials that have long been used as growth promoters in many countries (USA, Canada), as such tetracyclines, penicillins and sulfonamides [74].

*E. coli* is an important enteric and opportunistic pathogen of both humans and animals. *E. coli* infected animals develop clinical signs including enteritis, salpingitis, cellulitis, mastitis and septicemia [75]. Some strains of *E. coli* appear to be species specific, while others are responsible for clinical infection in humans. AMR is a rapidly growing concern for this zoonotic pathogen. ARB enters into the human food chain through inadequately treated drinking water [32] and consumed chicken meat [34], eggs and milk [24]. Wild birds [29], domestic scavenging poultry [17], commercial poultry farms [16] and contaminated environmental [52] interface all provide a reservoir for developing resistant bacteria. Unfortunately, resistance to third generation cephalosporins, fluroquinolones and/or carbapenems is increasing in developing countries as such Bangladesh.

Bangladesh, with its poor sewage [31] and water treatment facilities [30] exposes resistant bacteria to humans through their drinking water [32]. In addition, poor sanitation and personal hygiene also facilitates person to person transmission of enteric pathogens. All together, the globalised trade of animals and foods for increasing human consumption and the long distance migratory patterns of wildlife (birds), have lead to ARB being globally disseminated.

General measures are needed to address AMR in the context of global perspective, through controls on pollution from industrial, residential and agricultural sources. Innovative research as well as environmental monitoring and risk assessment is required to better understand the role of the environment in the selection and spread of AMR and there is an urgent need to strengthen the knowledge and evidence based research through surveillance and monitoring. Reducing the incidence of infection through effective sanitation, hygiene and infection prevention measures are of paramount importance. Overall, the basis is to optimize the use of antimicrobial medicines in human and animal health sectors as well.

### Limitations

1. Only a few numbers of animal species are included in this study as such domestic poultry, broiler and layer birds. Other species of livestock including cattle, sheep and goat are also important.
2. Only a few districts are within the coverage of prevalence determination.
3. We do not have sufficient publications or articles on residue concentrations and their impact on human health in foods consumed by humans, which have originated from livestock and aquaculture, so there is not enough data to make an interpretation regarding the risk of residue. There are no available guidelines regarding the concentrations of tolerable levels of antibiotics in food products.

## Conclusions

Future research should focus the following issues to gain better understanding the emergence of AMR issue and the ways to combat this public health threats in Bangladesh and global context as well:

1. The fate of ARB in the environment and wildlife is still unclear. Could AMR bacteria and mobile genetic elements carrying the resistance genes further evolve after their transfer to the environment? There are knowledge gaps regarding the magnitude and dynamic nature of spread regarding ARB and of ARGs within and between different ecological niches on farms, which deserve to be considered when assessing the transmission of ABR along the food chain.
2. Does the population of non-human ARGs have any relationship with the resistance population of human antibiotics? More research focusing on the human-animal-environmental interface and using novel approaches is required to understand the role of wild animals in the transmission of ABR and to assess potential risks for public health. A large-scale study of the Chinese poultry production system revealed a high prevalence of NDM-beta-lactamase-producing bacteria co-harbouring the plasmid-mediated colistin resistance gene *mcr-1* in poultry and also in insects and wild birds. This study pointed out that frequent dissemination of resistant isolates to the farm environment, is occurring and it highlighted the importance of wildlife in the further dissemination of high-risk bacteria that combine resistance to two groups of last-line antibiotics.
3. It is crucial to understand whether wild animals are just temporary carriers of the ARB or if the resistant bacteria obtained from the contaminated environment can be maintained in their gut for a long time, giving more opportunities for further transmission to other individuals in the population or through the environment to other species.
4. Steps to control antibiotic release and environmental disposal from all uses should be immediate and obligatory.
5. There is a need for detailed system biology analysis of resistance development in-situ.
6. Metagenomic analysis of bacterial pathogens from diverse sources including hospitals, veterinary clinics, agricultural sites, live animal processing markets and waste water plants, might underline the evolution of bacterial pathogens for integrin mediated resistance gene transfer in resistance evolution.
7. Standardisation of the surveillance methodology is essential. Regular surveillance needs to be conducted throughout the country to keep track of the resistance patterns of the pathogens.

## Methods

### Literature search strategy

An organized literature search approach was used to detect all published studies reporting prevalence of predominantly. *E. coli* and *Salmonella* spp., along with the use and resistance pattern of different antibiotics in Bangladesh. Google scholar, Pub Med, Science direct and Bangladeshi online journal platforms, such as Bangladesh Journals Online (www.banglajol.info) were searched for relevant studies published between 2005 and 2019. Specific Boolean words were developed based on the objectives of the study, as presented in Table 2. The search terms have been adopted into outcome, population, descriptive and area categories. The papers were downloaded using the CVASU library network. The Boolean words of each category were combined using “AND” whereas “OR” was used to join the term within a category. Some modification has been conducted based on the requirements of the search engines, and advanced search criteria has been used to search Google scholar.

**Table 2:** Electronic database search algorithm

Items	Boolean keywords
Outcome	Prevalence OR Incidence OR Occurrence OR Frequency OR Resistance OR Use OR Residues OR Identification OR Characterization OR Investigation OR Survey OR Rate
Population	E. coli OR Salmonella OR Antibiotics OR Antimicrobials
Descriptive	Domestic OR Street food OR Food OR Wildlife OR Insects OR Environment
Area	Barisal OR Chittagong OR Dhaka OR Mymensingh OR Rajshahi OR Rangpur OR Sylhet OR Bangladesh

### Data extraction and recording

A pre-tested data extraction spread sheet was developed to create the variable, in line with study objectives. Previous literature on systematic review and meta-analysis was used to evaluate the spread sheet. Data was extracted and recorded for study location, citation, first author, title, time of study, year of publication, type of specimen, sample size, number of positive specimens, amount of antibiotics, specific antibiotic sensitivity or resistance level percentages, methods of detection used, culturing techniques and resistant genes. The recorded data was categorized into food, domestic, environmental, wildlife and insects based on the nature of the samples.

### Data Analysis

All data was forwarded to STATA/IC-13.0 (Stata Corp, 4905 Lakeway Drive, College Station, Texas 77,845 USA) for statistical analysis. Descriptive statistics was done to identify the numbers of articles along with the percentages (%) and 95% CI. Study variations among the study were evaluated using Chi square (χ^2^) statistics followed by I^2^ statistics to determine the degree of heterogeneity in the study. Crude prevalence was estimated by dividing the positive number of samples by the total sample number. Standard Error (SE) was calculated using standard formula for proportion calculation. A random effect meta-analysis model was applied using ‘metan’ command specifying random due to the high degree of heterogeneity in the study (I^2^ > 75%). The output has been illustrated using a forest plot.

The antibiotic resistant percentage was calculated by using the number of samples tested which were resistant to specific antibiotics and contrasted with the total number of samples used for sensitivity testing. The result has been expressed as a percentage and illustrated in a proportionate bar diagram. As the antibiotics were grouped into their common genera some of the values are > 100% due to repeated estimation of antibiotics under each genera, as the previous literature did not state whether the samples were positive for more than one antibiotic or not.

## Data Availability

All data generated or analyzed during this study are included in this article.

## Abbreviations

AMR: Antimicrobial Resistance
ABR: Antibacterial Resistance
ARB: Antibiotic Resistance Bacteria
ARGs: Antibiotic Resistance Genes
AGF: Agricultural Food Products
CVASU: Chattogram Veterinary and Animal Sciences University
LMIC: Low and Middle-Income Countries
SEA: Southeast Asia
SE: Standard Error
CI: Confidence Interval

## References

[1] Chandler CIR (2019). Current accounts of antimicrobial resistance: stabilisation, individualisation and antibiotics as infrastructure. Palgrave Commun.

[2] Reygaert WC (2018). An overview of the antimicrobial resistance mechanisms of bacteria. AIMS Microbiol.

[3] Ahmed I, Rabbi MB, Sultana S (2019). Antibiotic resistance in Bangladesh: A systematic review. Int J Infect Dis.

[4] Byarugaba DK (2004). Antimicrobial resistance in developing countries and responsible risk factors. Int J Antimicrob Agents.

[5] Argudín MA, Deplano A, Meghraoui A, Dodemont M, Heinrichs A, Denis O, Nonhoff C, Roisin S (2017). Bacteria from animals as a pool of antimicrobial resistance genes. Antibiotics (Basel).

[6] Founou LL, Founou RC, Essack SY (2016). Antibiotic resistance in the food chain: A developing country-perspective. Front Microbiol.

[7] Shaikh S, Fatima J, Shakil S, Rizvi SM, Kamal MA (2015). Antibiotic resistance and extended spectrum beta-lactamases: Types, epidemiology and treatment. Saudi J biologic sci.

[8] Van Boeckel TP, Brower C, Gilbert M, Grenfell BT, Levin SA, Robinson TP, Teillant A, Laxminarayan R (2015). Global trends in antimicrobial use in food animals. PNAS.

[9] Rousham EK, Unicomb L, Islam MA (1876). Human, animal and environmental contributors to antibiotic resistance in low-resource settings: integrating behavioural, epidemiological and One Health approaches Proceedings of the Royal Society B. Biol Sci.

[10] World Health Organization. Joint. FAO/OIE/WHO Expert Workshop on Non-Human Antimicrobial Usage and Antimicrobial Resistance: scientific assessment: Geneva, December 1–5, 2003. World Health Organization 2004(WHO/CDS/CPE/ZFK/2004.7).

[11] Bengtsson-Palme J, Larsson DGJ (2015). Antibiotic resistance genes in the environment: prioritizing risks. Nature Rev Microbiol.

[12] Davies J, Davies D (2010). Origins and evolution of antibiotic resistance. Microbiol Mol Biol Rev.

[13] Akond MA, Alam S, Hassan SM, Shirin M (2009). Antibiotic resistance of *Escherichia coli* isolated from poultry and poultry environment of Bangladesh. Int J Food Safety.

[14] Begum K, Reza TA, Haque M, Hossain A, Hassan FK, Hasan SN, Akter N, Ahmed A, Barua U (2010). Isolation, identification and antibiotic resistance pattern of *Salmonella* spp. from chicken eggs, intestines and environmental samples. Bangl Pharm J.

[15] Ahaduzzaman M, Hassan MM, Alam M, Islam SK, Uddin I (2014). Antimicrobial resistance pattern against *Staphylococcus aureus* in environmental effluents. Res J Vet Pract.

[16] Hasan B, Faruque R, Drobni M, Waldenström J, Sadique A, Ahmed KU, Islam Z, Parvez MH, Olsen B, Alam M (2011). High prevalence of antibiotic resistance in pathogenic *Escherichia coli* from large-and small-scale poultry farms in Bangladesh. Avian Dis.

[17] Hasan B, Sandegren L, Melhus Â, Drobni M, Hernandez J, Waldenström J, Alam M, Olsen B (2012). Antimicrobial drug–resistant Escherichia coli in wild birds and free-range poultry, Bangladesh. Emerg Infect Dis.

[18] Mahmud MS, Bari ML, Hossain MA (2011). Prevalence of *Salmonella* serovars and antimicrobial resistance profiles in poultry of Savar area, Bangladesh. Foodborne Pathog Dis.

[19] Naurin S, Islam MA, Khatun MM (2012). Prevalence of *Salmonella* in apparently healthy chickens in Mymensingh. Bangladesh Microbes Health.

[20] Sultana F, Afroz H, Jahan A, Fakruddin M, Datta S (2014). Multi–antibiotic resistant bacteria in frozen food (ready to cook food) of animal origin sold in Dhaka, Bangladesh. Asian Pac J Trop Biomed.

[21] Mahmud T, Hassan MM, Alam M, Khan MM, Bari MS, Islam A (2016). Prevalence and multidrug-resistant pattern of *Salmonella* from the eggs and egg-storing trays of retail markets of Bangladesh. Int J One Health.

[22] Ahmed MM, Rahman MM, Mahbub KR, Wahiduzzaman M (2011). Characterization of antibiotic resistant *Salmonella* spp. isolated from chicken eggs of Dhaka city. J Sci Res.

[23] Marjan S, Das KK, Munshi SK, Noor R (2014). Drug-resistant bacterial pathogens in milk and some milk products. Nutr Food Sci.

[24] Uddin MA, Motazzim-ul-Haque HM, Noor R (2011). Isolation and identification of pathogenic *Escherichia coli*, *Klebsiella spp.* and *Staphylococcus spp.* in raw milk samples collected from different areas of Dhaka City, Bangladesh. Stamford J Microbiol.

[25] Hasan B, Islam K, Ahsan M, Hossain Z, Rashid M, Talukder B, Ahmed KU, Olsen B, Kashem MA (2014). Fecal carriage of multi-drug resistant and extended spectrum β-lactamases producing *E. coli* in household pigeons. Bangladesh Vet Microbiol.

[26] Dey RK, Khatun MM, Islam MA, Hossain MS (2013). Prevalence of multidrug resistant *Escherichia coli* in pigeon in Mymensingh. Bangladesh Microbes Health.

[27] Rahman MM, Hossain MK, Akhter MR, Hasan SM (2011). Characterization and antibiogram study of *Salmonella* serovars isolated from duck, quail and pigeon in Dinajpur district of Bangladesh. Int J Sustain Agric Tech.

[28] Hosain MS, Islam MA, Khatun MM, Dey RK (2012). Prevalence and antibiogram profiles of *Salmonella* isolated from pigeons in Mymensingh, Bangladesh. Microbes Health.

[29] Hasan B, Melhus Â, Sandegren L, Alam M, Olsen B (2014). The gull (*Chroicocephalus brunnicephalus*) as an environmental bioindicator and reservoir for antibiotic resistance on the coastlines of the Bay of Bengal. Microb Drug Resist.

[30] Adnan N, Sultana M, Islam OK, Nandi SP, Hossain MA (2013). Characterization of ciprofloxacin resistant extended spectrum β-lactamase (ESBL) producing *Escherichia spp.* from clinical waste water in Bangladesh. Adv Biosci Biotechnol.

[31] Akter F, Amin MR, Osman KT, Anwar MN, Karim MM, Hossain MA (2012). Ciprofloxacin-resistant *Escherichia coli* in hospital wastewater of Bangladesh and prediction of its mechanism of resistance. World J Microbiol Biotechnol.

[32] Talukdar PK, Rahman M, Rahman M, Nabi A, Islam Z, Hoque MM, Endtz HP, Islam MA (2013). Antimicrobial resistance, virulence factors and genetic diversity of *Escherichia coli* isolates from household water supply in Dhaka, Bangladesh. PloS ONE.

[33] Nandi SP, Sultana M, Hossain MA (2013). Prevalence and characterization of multidrug-resistant zoonotic *Enterobacter spp.* in poultry of Bangladesh. Foodborne Pathog Dis.

[34] Islam M, Sabrin MS, Kabir MHB, Karim SJI, Sikder T (2018). Prevalence of multidrug resistant (MDR) food-borne pathogens in raw chicken meat in Dhaka city, Bangladesh: an increasing food safety concern. Asian Australas J Biosci Biotechnol.

[35] Sobur MA, Sabuj AAM, Sarker R, Rahman AMMT, Kabir SML, Rahman MT (2019). Antibiotic-resistant *Escherichia coli* and *Salmonella* spp. associated with dairy cattle and farm environment having public health significance. Vet World.

[36] Ahmed S, Siddique MA, Rahman M, Bari ML, Ferdousi S (2019). A study on the prevalence of heavy metals, pesticides and microbial contaminants and antibiotics resistance pathogens in raw salad vegetables sold in Dhaka. Bangladesh Heliyon.

[37] Banik A, Abony M, Datta S, Towhid ST (2019). Microbiological quality of ready-to-eat food from Dhaka, Bangladesh. Curr Res Nutr Food Sci.

[38] Habibullah A, Rahman AM, Haydar MR, Nazir KH, Rahman MT (2017). Prevalence and molecular detection of methicillin-resistant *Staphylococcus aureus* from dogs and cats in Dhaka city. Bangl J Vet Med.

[39] Sarker MS, Mannan MS, Ali MY, Bayzid M, Ahad A, Bupasha ZB (2019). Antibiotic resistance of *Escherichia coli* isolated from broilers sold at live bird markets in Chattogram, Bangladesh. J Adv Vet Anim Res.

[40] Hassan MM, Begum S, Al Faruq A, Alam M, Mahmud T, Islam A (2018). Multidrug resistant *Salmonella* isolated from street foods in Chittagong, Bangladesh. Microbiol Res J Int.

[41] Faruq AA, Hassan MM, Uddin MM, Rahman ML, Rakib TM, Alam M, Islam A (2016). Prevalence and multidrug resistance pattern of *Salmonella* isolated from resident wild birds of Bangladesh. Int J One Health.

[42] Islam A, Nath AD, Islam K, Islam S, Chakma S, Hossain MB, Al-Faruq A, Hassan MM (2016). Isolation, identification and antimicrobial resistance profile of *Staphylococcus aureus* in cockroaches (*Periplaneta americana*). J Adv Vet Anim Res.

[43] Haque MA, Jewel MA, Al Masud A, Rahman MS, Hasan J (2018). Assessment of bacterial pollution in sediment of padma river, Rajshahi, Bangladesh. Curr World Environ.

[44] Tanzin T, Nazir KN, Zahan MN, Parvej MS, Zesmin K, Rahman MT (2016). Antibiotic resistance profile of bacteria isolated from raw milk samples of cattle and buffaloes. J Adv Vet Anim Res.

[45] Saifullah MK, Mamun MM, Rubayet RM, Nazir KN, Zesmin K, Rahman MT (2016). Molecular detection of *Salmonella* spp. isolated from apparently healthy pigeon in Mymensingh, Bangladesh and their antibiotic resistance pattern. J Adv Vet Anim Res.

[46] Sobur A, Haque ZF, Sabuj AAM, Levy S, Rahman AMMT, El Zowalaty ME, Rahman T (2019). Molecular detection of multidrug and colistin-resistant *Escherichia coli* isolated from houseflies in various environmental settings. Future Microbiol.

[47] Sobur A, Hasan M, Haque E, Mridul AI, Noreddin A, El Zowalaty ME, Rahman T (2019). Molecular detection and antibiotyping of multidrug-resistant *Salmonella* isolated from houseflies in a fish market. Pathogens.

[48] Rahman MM, Rahman MM, Meher MM, Khan MSI, Anower AKMM (2016). Isolation and antibiogram of *Salmonella* spp. from duck and pigeon in Dinajpur, Bangladesh. J Adv Vet Anim Res.

[49] Rahman SMR, Uddin MN, Nain Z, Karim MM (2019). Screening for microbial load and antibiotic resistance pattern in *Escherichia coli* isolated from paper currency circulating in Kushtia, Bangladesh. Int J Res Med Sci.

[50] Rahman MM, Amin KB, Rahman SMM, Khair A, Rahman M, Hossain A, Rahman AKMA, Parvez MS, Miura N, Alam MM (2018). Investigation of methicillin-resistant *Staphylococcus aureus* among clinical isolates from humans and animals by culture methods and multiplex PCR. BMC Vet Res.

[51] Hasan B, Olsen B, Alam A, Akter L, Melhus Â (2015). Dissemination of the multidrug-resistant extended-spectrum β-lactamase-producing *Escherichia coli* O25b-ST131 clone and the role of house crow (*Corvus splendens*) foraging on hospital waste in Bangladesh. Clin Microbiol Infect.

[52] Rashid M, Rakib MM, Hasan B (2015). Antimicrobial-resistant and ESBL-producing *Escherichia coli* in different ecological niches in Bangladesh. Infect Eco Epidem.

[53] Sarker MS, Ahad A, Ghosh SK, Mannan MS, Sen A, Islam S, Bayzid M, Bupasha ZB (2019). Antibiotic-resistant *Escherichia coli* in deer and nearby water sources at safari parks in Bangladesh. Vet World.

[54] Al-Salauddin AS, Hossain MF, Dutta A, Mahmud S, Islam MS, Saha S, Kabir SL (2015). Isolation, identification and antibiogram studies of *Salmonella* species and *Escherichia coli* from broiler meat in some selected areas of Bangladesh. Int J Basic Clin Pharmacol.

[55] Jahan M, Rahman M, Parvej MS, Chowdhury SM, Haque ME, Talukder MA, Ahmed S (2015). Isolation and characterization of *Staphylococcus aureus* from raw cow milk in Bangladesh. J Adv Vet Anim Res.

[56] Seel SK, Kabir SML, Islam MA (2016). Molecular detection and characterization of *Salmonella* spp. isolated from fresh fishes sold in selected upazila markets of Bangladesh. Bangl J Vet Med.

[57] Islam MK, Kabir SL, Haque AZ, Sarker YA, Sikder MH (2018). Molecular detection and characterization of *Escherichia coli*, *Salmonella* spp. and *Campylobacter* spp. isolated from broiler meat in Jamalpur, Tangail, Netrokona and Kishoreganj districts of Bangladesh. Afr J Microbiol Res.

[58] Ramey AM, Ahlstrom CA (2020). Antibiotic resistant bacteria in wildlife: Perspectives on trends, acquisition and dissemination, data gaps, and future directions. J Wildl Dis.

[59] Dolejska M, Literak I (2019). Wildlife is overlooked in the epidemiology of medically important antibiotic-resistant bacteria. Antimicrob Agents Chemother.

[60] Sattar S, Hassan MM, Islam SKMA, Alam M, Faruk MSA, Chowdhury S, Saifuddin AKM (2014). Antibiotic residues in broiler and layer meat in Chittagong district of Bangladesh. Vet World.

[61] Chowdhury S, Hassan MM, Alam M, Sattar S, Bari MS, Saifuddin AKM, Hoque MA (2015). Antibiotic residues in milk and eggs of commercial and local farms at Chittagong, Bangladesh. Vet World.

[62] Hossain MM, Barman AKA, Rahim MM, Hassan MT, Begum M, Bhattacharjee D (2018). Oxytetracycline residues in Thai pangas (*Pangasianodon hypophthalmus*) sampled from Sylhet sadar upazila, Bangladesh. Bangl J Zool.

[63] Grami R, Mansour W, Mehri W, Bouallègue O, Boujaâfar N, Madec J, Haenni M (2016). Impact of food animal trade on the spread of mcr-1-mediated colistin resistance, Tunisia, July 2015. Euro Surveill.

[64] McEwen SA, Collignon PJ. Antimicrobial Resistance: a One Health Perspective. Microbiol Spectr. 2018;6(2). 10.1128/microbiolspec.ARBA-0009-2017. 10.1128/microbiolspec.arba-0009-2017 PMC11633550 29600770

[65] Fick J, Söderstörm H, Lindberg RH, Phan C, Tysklind M, Larsson DGJ (2009). Contamination of surface, ground, and drinking water from pharmaceutical production. Environ Toxicol Chem.

[66] Ruuskanen M, Muurinen J, Meierjohan A, Pärnänen K, Tamminen M, Lyra C, Kronberg L, Virta M (2016). Fertilizing with animal manure disseminates antibiotic resistance genes to the farm environment. J Environ Qual.

[67] Singer AC, Shaw H, Rhodes V, Hart A (2016). Review of antimicrobial resistance in the environment and its relevance to environmental regulators. Front Microbiol.

[68] McEwen SA, Fedorka-Cray PJ (2002). Antimicrobial use and resistance in animals. Clin Infect Dis.

[69] van den Bogaard AE, Stobberingh EE (2000). Epidemiology of resistance to antibiotics: links between animals and humans. Int J Antimicrob Agents.

[70] Wales AD, Davies RH (2015). Co-selection of resistance to antibiotics, biocides and heavy metals, and its relevance to foodborne pathogens. Antibiotics.

[71] Aarestrup FM, Wegener HC, Collignon P (2008). Resistance in bacteria of the food chain: epidemiology and control strategies. Exp Rev Anti-infect Ther.

[72] Dutil L, Irwin R, Finley R, Ng LK, Avery B, Boerlin P, Bourgault AM, Cole L, Daignault D, Desruisseau A, Demczuk W, Hoang L, Horsman GB, Ismail J, Jamieson F, Maki A, Pacagnella A, Pillai DR (2010). Ceftiofur resistance in *Salmonella enterica* serovar Heidelberg from chicken meat and humans, Canada. Emerg Infect Dis.

[73] Chiu C-H, Wu T-L, Su L-H, Chu C, Chia J-H, Kuo A-J, Chien M-S, Lin T-Y (2002). The emergence in Taiwan of fluoroquinolone resistance in *Salmonella enterica* serotype Choleraesuis. N Engl J Med.

[74] World Health Organization (1997). The Medical impact of the use of antimicrobials in food animals: report of a WHO meeting, Berlin, Germany, 13–17 October 1997.

[75] Mellata M (2013). Human and avian extraintestinal pathogenic *Escherichia coli*: infections, zoonotic risks, and antibiotic resistance trends. Foodborne Pathog Dis.

